# Exploring Parent Perspectives of Feasibility After Intensive Telerehabilitation for Children with Unilateral Cerebral Palsy: A Mixed Methods Study

**DOI:** 10.1007/s10882-025-10033-9

**Published:** 2025-09-01

**Authors:** Elizabeth Maus, Angela Rittler, Kylie Amon, Jill Heathcock, Warren Lo, Stephanie DeLuca, Amy Darragh

**Affiliations:** 1 The Ohio State University, Columbus, OH USA; 2 Nationwide Children’s Hospital, Columbus, OH USA; 3 Fralin Biomedical Research Institute at Virginia Tech Carilion School of Medicine, Roanoke, VA USA; 4 Virginia Commonwealth University, Richmond, VA USA

**Keywords:** Constraint Induced Movement Therapy, Occupational Therapy, Physical Therapy, Qualitative, Telerehabilitation

## Abstract

To test the feasibility, acceptability, and appropriateness of parent-enacted telehealth pediatric constraint induced movement therapy (pCIMT) for children with unilateral cerebral palsy (CP). A pCIMT intervention was adapted for telehealth. Participants (*n* = 12) received 20 sessions (daily, 3 h each) of telehealth pCIMT over 4 weeks. Quantitative data was collected using the Feasibility, Acceptability, and Appropriateness of Intervention Measure (FIM, AIM, IAM) and Usefulness, Usability and Desirability (UUD). Qualitative data was collected via semi-structured, post-treatment, parent interviews and coded using inductive content analysis and constant comparison. Quantitative and qualitative results support feasibility, acceptability, and appropriateness of telehealth pCIMT and usefulness, usability, and desirability of study technology. Median scores on Feasibility, Acceptability, and Appropriateness of Intervention Measure (out of 20 possible points) were 20 (IQR = 5.75), 18.5 (IQR = 3.75) and 20 (IQR = 4), respectively; median ratings on the Usefulness, Usability and Desirability Assessment (7 point likert scale 1–7) were 6 (IQR = 1), 6.5 (IQR = 1), and 6 (IQR = 2.5) respectively. Content analysis of parent interviews yielded three categories: Implementation Potential, Advantages and Disadvantages, and Logistical Considerations. Telehealth pCIMT was feasible, acceptable, and appropriate but is not without challenges. A demonstration of the efficacy of telehealth pCIMT is needed. Registered on Clinicaltrials.gov on 3/21/2022 with ID NCT05303883.

**Supplementary Information:**

The online version contains supplementary material available at 10.1007/s10882-025-10033-9.

## Introduction

Daily Pediatric Constraint Induced Movement Therapy (pCIMT) is a known efficacious motor intervention for children with unilateral cerebral palsy (CP), yet clinical- and family-level barriers impact translation into practice (Hoare et al., 2019; Novak et al., 2020). Clinical barriers include lack of training, staffing levels, and funding for intensive programs, which create barriers for scheduling and delivering effective doses of pCIMT (McConnell et al., 2012). When families do not live near a pCIMT program or do not have the ability to travel extended distances there is an access gap (Marcin et al., 2016; Teleman et al., 2021). Children who live nearby or can travel to pCIMT programs may complete multiple bouts of pCIMT during childhood, while children who live far from programs may never have access (Charles & Gordon, 2007; DeLuca et al., 2015). Travel time alone can prevent participation in pCIMT, particularly in rural and under-resourced communities (Hirko et al., 2020; Marcin et al., 2016). This creates health disparity.

Telehealth-delivered, parent-implemented, constraint-induced movement therapy (pCIMT) may help address access barriers for children with unilateral CP, particularly in families unable to attend in-person therapy. While feasibility and preliminary efficacy have been demonstrated in adults post-stroke and infants under 3 years old (Cramer et al., 2019; Hurd et al., 2024; Svensson et al., 2024), it is not yet clear whether telehealth pCIMT is feasible or appropriate for a broader range of young children. In this developmental stage, children often exhibit partial functional independence and may resist performing tasks with the more affected upper extremity (Burgess et al., 2019; Hoare et al., 2019). Because home-based telehealth motor interventions rely heavily on the family unit, the role of the parent becomes especially important. In telehealth protocols, therapists guide parents to enact the intervention, planning and administering activities in the home, which differs from traditional models where therapists provide direct intervention (Camden et al., 2020). “Parent enactment” captures this dynamic most accurately, emphasizing parents’ learning and application of therapeutic strategies with their child (Piotrowska et al., 2017). Parent-enactment models are well established for in-person pediatric rehabilitation (Akhbari Ziegler & Hadders-Algra, 2020; Alonazi, 2021; Kaur et al., 2022; Lord et al., 2018). For infants and preschoolers under 3.5 years old, parent-enacted pCIMT has resulted in improved upper extremity function (Eliasson et al., 2018; Shierk et al., 2023; Svensson et al., 2024). However, whether this success can be extended to older or more independent children remains unknown. Given these complexities, it is essential to evaluate the feasibility of telehealth pCIMT using both quantitative and qualitative methods to better understand the parent perspective and identify factors that may support or hinder implementation before proceeding to efficacy trials.

Feasibility studies often precede larger efficacy trials to identify elements that are critical to the success of a larger trial (Aschbrenner et al., 2022; Eldridge et al., 2016). A *mixed-methods* feasibility study provides rich insight into key barriers and facilitators (Bowen et al., 2009). This mixed methods feasibility study, therefore, sought to assess the feasibility, acceptability, and appropriateness of a parent-enacted, telehealth version of the CHAMP protocol (Ramey et al., 2021). Specifically, this study, CHAMP-Telehealth (CHAMP-T), assessed adherence to intervention dose, parent perceptions of the technology used during telehealth, and barriers and facilitators to the intervention and assessment processes.

## Methods

### Participants

Participants (*n* = 12) with unilateral CP were enrolled in this study. Study participants met the following inclusion criteria: 1) ages 1–10 years old at enrollment; 2) diagnosis of unilateral CP; and 3) ability to participate in therapy three hours per day, five days per week for four weeks. In addition, each child needed one or more adult caregivers committed to participating in therapy three hours per day alongside the child. Children with uncontrolled seizures or other medical conditions that precluded tolerance of three hours of therapy per day were excluded. Participants received intervention free of charge and an additional renumeration of $75 per completed assessment, for a total of $150.

Participants were recruited between May 2022-October 2023 through word of mouth, social media, and local clinics. The trial was registered on ClinicalTrials.gov (NCT05303883). The Ohio State University Institutional Review Board approved the study. All families completed informed consent and signed Parent Permission forms. Verbal assent from the child was completed when age allowed.

### Study Design

This study used a single group, convergent, mixed methods design (Creswell & Plano Clark, 2017), in which quantitative and qualitative data were collected separately but during the same overall study period, analyzed independently, and integrated during interpretation to provide a more comprehensive understanding of parent experiences with the CHAMP-T program. Qualitative methodology is described in *Data Analysis* and *Data Trustworthiness* sections below.

### Measures

**Fig. 1:**
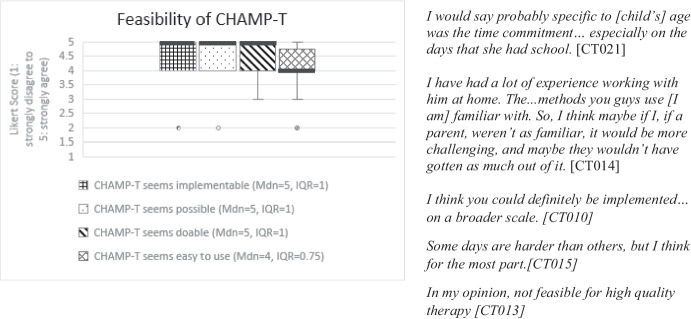
Box and whiskers plot of individual items (x-axis) and median FIM score (y-axis). Median scores are designated by a horizontal line. ”Seems doable” and “seems easy to use” show the greatest variability (whiskers) in scores. Representative quotes are included to the right of the bar graph and Table 3

**Fig. 2:**
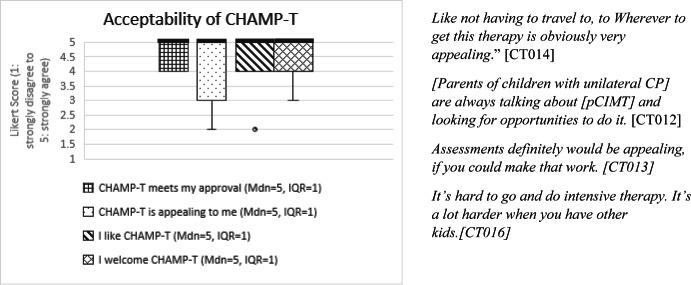
Box and whiskers plot of individual items (x-axis) and median AIM score (y-axis). Median scores are designated by a horizontal line. Representative quotes are included to the right of the bar graph and Table 3

**Fig. 3:**
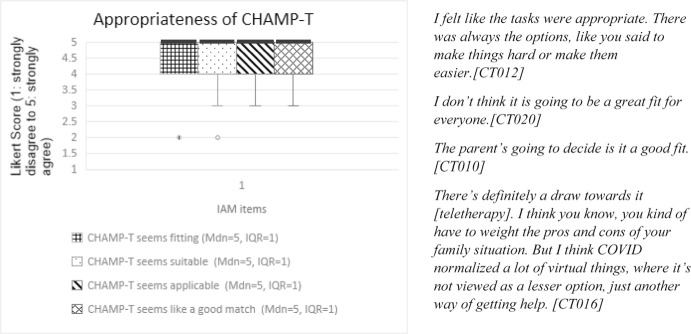
Box and whiskers plot of individual items (x-axis) and median IAM score (y-axis). Median scores are designated by a horizontal within the box plot. Representative quotes are included to the right of the bar graph and also in Table 3

Parents of all participants completed a baseline demographic questionnaire about their child and family as well as pre- and post-intervention assessment (described below under *Telehealth Assessment*). After the intervention period and post-intervention assessment, parents were emailed electronic copies of the Feasibility of the Intervention Measure, Acceptability of the Intervention Measure, and Appropriateness of the Intervention Measure (FIM, AIM, IAM) and the Usefulness, Usability and Desirability (UUD) to complete and return via email (Darragh et al., 2016; Weiner et al., 2017). Notably, when the FIM is discussed in this paper, it refers to the Feasibility Intervention Measure and not the Functional Independence Measure, which shares the same abbreviation. The FIM, AIM, and IAM are scored on a 5-point Likert Scale (1–5) while the UUD is scored on a 7-point Likert Scale (1–7). These tools and their distinct constructs are described in Table 1. Item level verbiage for both tools was adapted to be specific to the CHAMP-T intervention while maintaining the key construct (feasibility, acceptability, appropriateness, usefulness, usability or desirability) (Tables 1). For example, instead of a question from the UUD reading, “I find this learning activity too complicated,” it was adapted to “The CHAMP-T technology was too complicated.” All questions from the FIM, AIM, IAM (Figs. 1, 2 and 3) and UUD (Table 4) were adapted in this way. Negatively worded UUD items (usability *n* = 4, usefulness *n* = 3, desirability *n* = 2) were reverse coded. Part of feasibility included adherence to the dosage, as measured by treatment session documentation. These quantitative data were analyzed prior to qualitative analysis using descriptive statistics (e.g., frequencies, means, medians) to characterize responses to each measure and identify patterns across participants.

**Table 1:** Quantitative CHAMP-T outcome measures

Construct	Definition and components	Measurement tool	# items	Measurement scale
Feasibility	*Adherence* to dosage (measured by number of minutes per day, days per week, and treatment weeks completed for each child)	Treatment session notes	N/A	N/A
	*Ease *of treatment delivery and assessment procedures, including barriers to and supports for broader implementation in a clinical trial	Feasibility of Intervention Measure (FIM)	4 items	1: strongly disagree to 5: strongly agree
Acceptability	The extent to which the parent *approved of* remote delivery and found it *appealing* and *desirable*	Acceptability of Intervention Measure (AIM)	4 items	1: strongly disagree to 5: strongly agree
Appropriateness	The *fit* or *match* between the treatment and assessment and the remote delivery	Intervention Appropriateness Measure (IAM)	4 items	1: strongly disagree to 5: strongly agree
Usability	*Ease of use* of the telehealth technology	Usefulness, Usability, and Desirability (UUD)	10 total items	Items: 1: strongly disagree to 5: strongly agree Overall score: 1: not usable at all to 7: very usable
Usefulness	*Value* and *applicability* of the telehealth technology	Usefulness, Usability, and Desirability (UUD)	6 items	Items: 1: strongly disagree to 5: strongly agree Overall score: 1: not useful at all to 7: very useful
Desirability	*Overall appeal* or *interest* in the telehealth technology	Usefulness, Usability, and Desirability (UUD)	5 items	Items: 1: strongly disagree to 5: strongly agree Overall score: 1: do not like to 7: like it very much

Quantitative CHAMP-T outcome measures included adherence to dosage, the Feasibility of Intervention Measure (FIM), Acceptability of Intervention Measure (AIM), and Intervention Appropriateness Measure (IAM) (Weiner et. al, 2017) and Usefulness, Usability and Desirability (UUD) (Darragh et. al, 2016)

#### Post-Treatment Interview

Post-treatment, all parents also completed a 30–60 min semi-structured interview with trained study staff via Microsoft Teams. Parent interviews were scheduled within 2 weeks of concluding treatment. All interviews followed a consistent interview guide (See Supplementary File S1). Interview questions covered topics such as ease of completing remote sessions, perceived effectiveness of remote delivery, generalizability of the approach, benefits and drawbacks for families and providers, and suggestions for improvement. Probing questions were used to explore barriers and facilitators to participation in telehealth pCIMT, especially for families facing challenges such as geographic location, travel difficulties, or schedule limitations. Interviews were video and audio recorded, transcribed verbatim, and verified.

### Procedures

#### Supplies

Several items were shipped to the participant’s home for study participation, including 1) study technology, 2) a constraint for the unaffected upper extremity, and 3) materials for intervention and assessment. Technology supplies included a Kubi® Telepresence Robot stand and iPad. The Kubi® robot was controlled remotely by the therapist to tilt, pan, and adjust the view (Reifenberg et al., 2017). The soft, removable constraint was a prefabricated splint, sized for the child. Assessment materials included the test kit for the Box and Blocks Test (BBT) (Mathiowetz et al., 1985), a coloring page, and a printed copy of the ABILHAND-KIDS (Arnould et al., 2004). Individualized toys (e.g., board games, craft supplies) were shipped to the family for use during the intervention to promote engagement. Families kept these toys. A return shipping label was provided for each family to send the technology, constraint, and assessment tools back to the study site following the completion of the study.

#### Pre-Treatment Meeting

Before the assessment, parents were trained in setting up and using the Kubi® robot and iPad during a 10–15 min videoconferencing session. This included identifying a stable surface where the Kubi® and iPad could be placed to visualize the participant during treatment, connecting the iPad to the home’s WiFi and pairing the Kubi® to the iPad via Bluetooth. Intervention goals were developed with the parents and child (as appropriate) prior to the start of treatment. Intervention-related training on principles of motor and behavioral learning were integrated throughout each synchronous intervention session.

#### Intervention

The CHAMP-T intervention was delivered via Microsoft Teams. Treatment sessions were recorded and stored on a password-protected, secure network. The synchronous therapist-directed sessions, integrated principles of family-centered care to facilitate confidence with parent-enactment (Novak et al., 2020; King et al., 2017; Wallisch et al., 2019). The intervention was delivered three hours per day, five days per week for four weeks, for a total dose of 180 min each day or 3600 min in 4 weeks. The intervention was delivered in two components 1) 120 min per day of synchronous, therapist-directed instruction via telehealth and 2) 60 min per day of asynchronous, parent-enacted treatment activities. During synchronous time, the therapist, child, and parent met via a secure Microsoft Teams video conference. During the asynchronous time, the parent and child practiced play and routine-based therapeutic activities together, without the therapist. Because the parent is delivering therapist-designed activities both during synchronous and asynchronous time, the entire CHAMP-T intervention can be considered therapist-directed and parent-enacted. The distinction between the two components is whether the intervention was synchronous with therapist, parent, and child or asynchronous with only parent and child. The therapist documented adherence to dosage of synchronous sessions using Microsoft Teams timestamps and dosage of asynchronous sessions using parent report. These values were documented within the daily treatment note. A full intervention period was defined as 17 out of 20 intervention days, equivalent to 3,060 min (Ramey et al., 2019). CHAMP-T was modeled on the CHAMP protocol (Ramey et al., 2021) to include similar components, including child-centered activities, self-generated movements, and behavioral learning principles (e.g., operant conditioning).

#### Telehealth Assessment

To thoroughly assess the feasibility of multiple telehealth assessment options, three types of assessment were used: 1) a performance-based, standardized assessment, the Box and Blocks Test, which was administered by the parent and scored by the therapist remotely during a synchronous session on Microsoft Teams, 2) a parent-reported outcome, the ABILHAND-KIDS, and 3) a structured play and self-care activity session designed by the study team. Assessment-specific feasibility is summarized in the results. All assessments were completed within two weeks of concluding treatment.

### Data Analysis

Quantitative data were analyzed descriptively to identify adherence to full intervention dose (e.g. session length, number of sessions, session breaks, asynchronous parent-enacted activities). Scores from the FIM, AIM, IAM, and UUD were analyzed using medians and IQR (Table 1). Qualitative analysis was completed in Dedoose v9.0. Interview transcripts were analyzed using inductive content analysis (Elo & Kyngäs, 2008) to identify recurring patterns related to parent experiences with CHAMP-T. We applied a constant comparison method (Korstjens & Moser, 2017) and instrumental case study methodology (Baxter & Jack, 2008) in which segments of text were continuously compared within and across transcripts to refine codes into categories and sub-categories. Two independent coders analyzed the data and resolved discrepancies through discussion. Integration of quantitative and qualitative data occurs in the *Discussion* section below. Triangulation of results in this way allows for a richer interpretation of parent experiences and highlighted areas of convergence and divergence across data sources.

### Data Trustworthiness

The analysis team included a senior researcher, two doctorally-trained therapists (physical and occupational therapist), and an undergraduate student. To ensure data credibility, the research team used triangulation of analysts and each interview was coded independently by two team members to enhance the credibility and trustworthiness of the findings (Creswell & Plano Clark, 2017). Bracketing techniques were used throughout coding to minimize bias, and all analytic decisions were documented. Open codes were stored outside of Dedoose, while final coding was maintained within the platform. Although Dedoose offers interrater reliability metrics, we prioritized consensus and transparency over statistical agreement. The final coding structure was confirmed by the entire analysis team in a consensus meeting. Code categories and subcategories were considered trustworthy when supported by multiple participants, and negative case analysis was used to test for disconfirming evidence and ensure representativeness with multiple voices supported each.

## Results

Eleven participants completed the CHAMP-T study including baseline assessment, 4 weeks of treatment, post-assessment, and semi-structured interview. Median time between the conclusion of treatment and interview was 7 days (IQR = 4.25). Notably 2/10 interviews (CT010 and CT013) were rescheduled and occurred 1–3 months post-intervention. One child dropped out part-way through the treatment period, citing a change in family schedule. Most children (91.7%) were between the ages of 3-8 years old, attended school during the treatment period, had at least one sibling at home, and had at least two adults living in the home. Parents were classified as working (*n* = 5, 41.6%) or stay at home (*n* = 7, 58.3%). The types of goals set by families included self-help skills (e.g., dressing), eating and drinking (e.g., opening a snack package, holding a cup), and increasing the use of the affected hand during everyday UE activities. Participant demographics are outlined in Table 2.

**Table 2:** Participant characteristics

Baseline characteristic	*N* (%)
Sex	
Male	7 (58.3)
Female	5 (41.7)
Prematurity	
Yes	3 (25.0)
No	9 (75.0)
Range (weeks)	29 - 30
Ethnicity	
Non-Hispanic or Latino	11 (91.7)
Not Reported	1 (8.3)
Race	
White	11 (91.7)
Not Reported	1 (8.3)
Seizure Disorder	
Yes	4 (33.3)
No	8 (66.7)
Affected Upper Extremity	
Right	10 (83.3)
Left	2 (16.7)
History of pCIMT	
Prior pCIMT	6 (50.0%)
No prior pCIMT	6 (50.0%)
School Attendance	
Attended school	8 (66.7)
School Break	2 (16.6)
No school	2 (16.6)
Siblings	
No	2
Yes	10
Adults Living in Household	
2	11
3 or more	1
Part-time, in-home help (e.g., nanny, grandparents)	4
	*mean* (Range)
Age (months)	
Age (baseline)	63.3 (21–96)
Age at first pCIMT^a^(*n* = 6)	20.6 (12 - 36)
Age at most recent pCIMT^b^ (*n* = 6)	56.2 (48 - 60)

Baseline characteristics for *n* = 12 subjects enrolled in CHAMP-T. ^a,^average age of first exposure to pCIMT; ^b^average age at most recent exposure to pCIMT among those (n=6) with a history of pCIMT

Quantitative data is presented as medians of FIM, AIM, IAM and UUD in Tables 3 and 4 and as means and percentages for adherence to intervention dose and assessment procedures. Content analysis revealed three categories: Implementation Potential, Advantages and Disadvantages, and Logistical Considerations. Quantitative and qualitative data were integrated within the Implementation Potential category. Advantages and Disadvantages and Logistical Considerations reflect qualitative data only, as corresponding constructs were not captured in the quantitative measures.

**Table 3:** Quantitative and qualitative results of feasibility, acceptability, and appropriateness of CHAMP-T

Construct	Quantitative results	Qualitative quotes	Interpretation/integration
Feasibility	Median FIM = 20/20 (IQR = 5.75)	*“I think it’s gonna be difficult for whoever does it, but I think it is doable as long as you’re willing and willing to put in the time and effort to do it.” (CT020)*	High quantitative feasibility from adherence data and FIM are supported by qualitative reports
	82% received ≥ 90% dose Mean daily synchronous dose = 115.52 min (SD = 5.72); Mean daily asynchronous dose = 52.41 min (SD = 18.91)	*“Maybe spread it out a little bit and just do four days a week.” (CT015)* *“Maybe… two or three weeks even.” (CT014)*	Parents note CHAMP-T was challenging but feasible and suggest dose adjustments may improve fit
Acceptability	Median AIM = 18.5/20 (IQR = 3.75)	*“Like not having to travel… is obviously very appealing.”* (CT014)	High acceptability consistent with valuing telehealth benefits and reduced logistical burdens
		*“It’s hard to do intensive therapy… when you have other kids.”* (CT016)	Telehealth as an attractive alternative amid competing family demands
Appropriateness	Median IAM = 20/20 (IQR = 4)	*“The repetitions… I think that is a good match for teletherapy.”* (CT014) *“I don’t think it’s going to be a great fit for everyone.”* (CT020)	Quantitative and qualitative data show telehealth suitability and may fit some family systems more than others
		*“[on a screen] you can’t see exactly the movements that you’re doing… it’s okay for adults, but not kids.” (CT013)*	Telehealth works better for some than others. Investigation of child/family factors that impact suitability is needed

**Table 4:** CHAMP-T usability, usefulness and desirability

Indicators		Median (IQR)	Quote
Usability			
	1.Easy to Use	5 (1)	*“I think the remote part was as far as like the technology goes was easy and communicating with you [therapist] was easy.” [CT020]* *“You’re limited to a certain area in your house and in your space…couldn’t necessarily take it outside and get a connection.” [CT010]*
	2.Too complicated^a^	1.5 (1)	
	3.Took too long to learn^a^	1 (0.75)	
	4.Easy to complete activities	5 (1)	
	5.Easy to communicate with the therapist	*4.5 (1.75)*	
	6.Hard to see the therapist^a^	*2 (1.5)*	
	7.Easy to adjust the robot	4 (1.5)	
	8.Easy to connect	4.5 (1)	
	9.Other families would need help^a^	2.5 (1)	
	10.Families would learn to use quickly	4 (1)	
	**Overall Usability Rating (1–7)**	**6 (1)**	
Usefulness			
	1.Helpful for doing therapy	*4 (1)*	*“The Kubi is probably the best helper for this type of therapy.” [CT021]* *“We noticed the signal, would get funky, and if you were trying to do [therapy] at whatever [different] place.” [CT013]*
	2.Useful for communicating with therapist	*5 (1)*	
	3.Did not help implement therapy/assessment^a^	*1 (1)*	
	4.Robot was helpful for receiving remote therapy/assessment	*5 (1)*	
	5.Was not useful during therapy/assessment^a^	2 (1)	
	6.Didn’t help me use CHAMP-T therapies/assessment with my child^a^	2 (1)	
	**Overall Usefulness Rating (1–7)**	6.5 (1)	
Desirability			
	1.I liked the iPad	4.5 (1)	*“[The child] liked the Kubi robot, he thought it was interesting, so in that way it had added like a little pizzazz.” [CT012]* *“When she was done with something or mad about something, she just wanted to walk away because she knew you couldn’t see her.” [CT018]*
	2.I liked the robot	5 (1)	
	3.I wish this was available for more families	5 (1)	
	4.I didn’t like the remote delivery of CHAMP-T^a^	1.5 (1)	
	5.I didn’t like communicating with the therapist using the tech^a^	1.5 (1)	
	**Overall Desirability Rating (1–7)**	6 (2.5)	

^a^Indicates negatively worded UUD items (usability *n* = 4, usefulness *n*−3, desirability *n* = 2)

### Implementation Potential

Implementation potential is defined as the ability to deliver CHAMP-T, which is assessed through adherence to dosage and the FIM, AIM, IAM and UUD.

#### Feasibility of Intervention

Feasibility included whether CHAMP-T was implementable, possible, doable, and easy. Interview quotes representative of parent impressions of acceptability are included in Fig. 2 and Table 4. Median FIM total scores were high at 20/20 (IQR = 5.75). Individual FIM item-level scores were favorable (median = 5, IQR = 1). Statements from semi-structured interviews reflected that CHAMP-T intervention was doable and implementable but also room for improvements. Quotes from parent interviews related to feasibility are excerpted in Fig. 1 and Table 3.

Feasibility also included adherence to and ease of delivery of the CHAMP-T intervention and assessment as outlined in Table 1. The average daily synchronous therapist-directed dose was 115.52 min (SD = 5.72 min) and the average daily asynchronous parent-enacted dose was 52.41 min (SD = 18.91 min). Six of eleven participants (54.5%) met the minimum dose of at least 3,060 min over 4 weeks. Nine of eleven (81.8%) received at least 90% of the minimum dose (2,754–3,060 min). Doses less than 90% were due to 1) not completing asynchronous parent-enacted activities (*n* = 1), 2) inability to sustain two hours of synchronous therapist directed activity (*n* = 1).

#### Feasibility of Telehealth Assessment

Telehealth assessment feasibility using the Box and Blocks test and structured play session was limited: only 2 of 11 children (18%) completed the Box and Blocks Test at both time points due age, refusal, or broken/lost assessment equipment. Structured activity session recordings were largely unusable due to video framing issues in Microsoft Teams, and only 6 of 11 families returned both pre- and post-assessment ABILHAND forms.

#### Acceptability and Appropriateness

The acceptability of CHAMP-T included its appeal and likeability while appropriateness assessed the extent to which families described pCIMT as a good match for remote delivery. AIM and IAM total scores and item level scores are presented as medians and inter-quartile ranges on the left side of Table 3 and Figs. 2 and 3. In Fig. 2, acceptability items “CHAMP-T is appealing to me” and “I welcome CHAMP-T” show the greatest variability (whiskers) in scores. There is a clear outlier (score of “2”) for the item “I like CHAMP-T.” Item level scores on the IAM are also variable but the median is high at 5. Qualitative data, in the form of quotes from parent interviews, are presented in the center of Table 3 and on the right side of Figs. 2 and 3.

#### Usefulness, Usability, and Desirability

Through the UUD and semi-structured interviews, parents rated and commented on the usefulness, usability, and desirability of the CHAMP-T technology (Kubi® robot and iPad). Parent(s) of 10/11 subjects completed and returned the UUD; however, one parent omitted three questions about usefulness. Scores across all three constructs were positive (see Table 4) with higher usability scores than usefulness or desirability. Several challenges to consider with the use of CHAMP-T technology include: internet access, mobility throughout the home, and the limitations of the screen view.

### Advantages and Disadvantages

*Advantages* encompass the perceived benefits, including the natural environment and reduced travel and costs, while *disadvantages* include perceived challenges of CHAMP-T. No quantitative data on the advantages and disadvantages of CHAMP-T were collected. Therefore, only qualitative data is reported below. *Logistical Considerations* focus on the practical aspects needed to carry out CHAMP-T successfully. Quotes from parent interviews from each of these categories are provided in Table 5.

**Table 5:** Qualitative categories, sub-categories, and representative quotes

Category	Sub-category	Quote
Advantages	Natural environment	“There are things that we do every single day that we have in our life…I don't need to go purchase this tool or that tool or whatever. It's just stuff that we have, we can use and it's more meaningful.” [CT010] “The best part of remote intervention was that he could, like, continue to, like, go to school and do life and all that kind of stuff while still getting his therapy.” [CT014]
	Strength of CHAMP-T	“You wouldn’t have to find a babysitter or find transportation or time in your day.” [CT018]
	Access to expert in pCIMT	“One of the benefits of virtual is that you can have a specialist in hemiplegia regardless of where you live.” [CT012] “[Therapists] specialize in the conditions and treatments that the child actually needs rather than just being thrown with the most, the closest location.” [CT016]
	Carry over	“It's easier to be and it's also nice to be able to be involved so I can see what she does during therapy and try and mimic it.” [CT015]
Disadvantages	Demanding for parent	“It was hard to do everything all the time.” [CT019]
		“It was just more on me, not just throughout the day, but it was like very demanding during the treatment.” [CT016]
	Parent in role of therapist	“That was a little bit more challenging, I think mostly just because she's more resistant to me telling her how to do things versus like when she's in therapy.” [CT015]
		“[He] saw me as the person and he thought he could behave how he want. Maybe not put in as much effort
		“I don't think I got as much out of him as you could have done in person if I had left the room.” [CT013]
	In-Person vs. Virtual	“You [therapist], like, having your hands on him rather than mine because you know where to place, where your hand should be placed. And I don't… like where to help him and where not to help him.” [CT020]
Logistical Considerations	Preparation	“For parents, it’s very hard. It would, it would have to come with a disclaimer I think just to warn people, how hard it's going to be.” [CT013]
	Preparation	“I just wish, a little more like today these are the things these are the four activities we really want to focus on.” [CT019]
	Multiple Caregivers	“You kind of need another person to like push and you know, keep going for therapy and, you know, just kind of put up with his [child’s] uncomfortability at times.” [CT019]
	Future Improvements	“I think that would part of that pre visit would be, you know maybe walking around a room to room and like the therapist being like, ‘OK, like, you know, what are the available rooms? What do we have?’ and kind of prepping that out ahead of time.” [CT011]
	Schedule	“It would be harder for us during the school year” [CT015] “During school, I think it's better because she just gets all over with. [CT018] “Being able to be flexible with the time was really what helped a lot.” [CT016]

## Discussion

This feasibility study aimed to determine whether a pCIMT protocol (CHAMP-T) could be delivered via telehealth. The quantitative results clearly indicate that CHAMP-T was feasible, acceptable, and appropriate for parents of children with unilateral CP. Qualitative data aids in our understanding of the specific barriers and facilitators that made CHAMP-T feasible, acceptable and appropriate from the perspective of parents.

### Feasibility

Quantitative and qualitative data from this study support the feasibility of CHAMP-T. Several families identified that not needing to travel, whether locally, regionally, or nationally, was a facilitator. Traveling to medical appointments can be a source of stress and financial strain for families of children with complex healthcare needs, particularly when the distance traveled is more than 30 km (18.6 miles) or requires multiple days per week due to time off work, lost wages, and travel costs (Ballantyne et al., 2019; Christy et al., 2010; Hiscock et al., 2022). Telehealth programs, like CHAMP-T can be implemented from anywhere and may provide much-needed flexibility, convenience, and cost-savings to enable access to intensive interventions.

Although most families completed the intervention protocol, several parents reported it was demanding or suggested re-distribution of the dose. Long sessions are standard in intensive interventions, providing ample opportunities for repeated motor practice. (Bailes et al., 2008; Christy et al., 2010). In-person intensive programs face similar challenges– they are harder to fit into daily routines and balance with competing responsibilities such as work, school, and sibling activities (Christy et al., 2010; Hurd et al., 2024). Two recent infant telehealth protocols which required daily practice (supervised and unsupervised) successfully implemented a dose of 20–28 h distributed over 8–12 weeks (Beani et al., 2020; Schlichting et al., 2022). Given that older children require a higher dose of pCIMT intervention for treatment efficacy, a distributed dose in this age group may not be the answer (Jackman et al., 2020; Sakzewski et al., 2015). Balancing the delivery of intensive interventions with feasibility for families remains a challenge for in-person and telehealth models.

### Acceptability

Parents in CHAMP-T rated items on the AIM favorably. Interview quotes reflect appreciation for having autonomy to choose amongst intervention options based on what works best for their family and child. This aligns with broader research on telehealth in occupational and physical therapy, which recommends that discussing options and listening to preferences is ideal for meeting the needs of individual families (Lee et al., 2024; Proffitt et al., 2021). Many parents sought CHAMP-T because intensive pCIMT programs were unavailable locally (Felter et al., 2022; Palisano et al., 2012). This is also consistent with other studies, with one study reporting families traveling as far as 700 miles to participate in intensive therapy (Hall et al., 2023). Parents voiced that CHAMP-T was a preferred alternative to no therapy or non-intensive, in-person therapy. Conversely, the demands of intensive telehealth therapy, which requires parents to integrate therapist feedback on assistance, environment, and task difficulty while also managing their child’s emotional responses to challenging activities, must be carefully considered, as parents of children with CP face higher risks of stress and fatigue (Garip et al., 2017). Telehealth therapists could help mitigate this by supporting parents in behavior management as well as intervention principles (Lord et al., 2018; Whittingham et al., 2011). Further research is needed to explore how parent-enacted interventions affect parental workload and mental health.

### Appropriateness

Parents rated IAM items favorably and when interviewed, identified facilitators such as parent-therapist collaboration and feeling more involved in their child’s therapy, but there are also areas for improvement. In CHAMP-T, effective parent-enacted intervention was supported by a collaborative relationship, an essential element of effective parent-enacted intervention (Lord et al., 2018). For example, parents shared insights about their child’s motor performance or engagement while therapists applied motor learning principles and adapted tasks based on parent feedback. Some parents appreciated having greater control over therapy, being actively involved in the session, and spending dedicated one-on-one time with their child. These benefits align with several themes that are commonly reported in qualitative pediatric healthcare research including involvement, autonomy, and collaboration (Elangkovan & Shorey, 2020; Hayles et al., 2015). One barrier parents identified was enacting key components of motor learning such as task progression/regression, immediate feedback, and reinforcement. Resources and modules on these concepts, such as those described in prior studies may mitigate this barrier (Christie et al., 2022; Shierk et al., 2023). Further work on implementing intensive interventions and effective parent training to support enactment remain important priorities in pediatric rehabilitation.

### Technology

Similar to other telehealth studies (Schlichting et al., 2022; Svensson et al., 2024), families in CHAMP-T were successful in using the telehealth technology, with favorable UUD scores and positive feedback on the iPad, Kubi®, and Microsoft Teams platform. Parents appreciated features like therapist-controlled camera angles (Kubi®) and engaging virtual backgrounds (Teams), but some faced challenges with WiFi connectivity, outdoor use, and keeping devices charged. Reifenberg and colleagues (2017) reported similar difficulties with charging using the same technology. Technology-related challenges impacted both assessment quality and data completeness in CHAMP-T. Several children had difficulty completing the Box and Blocks Test due to engagement issues or damaged kits, and a technical error in video recordings prevented analysis of most structured play sessions. Digital versions of parent-reported measures (FIM, AIM, IAM, UUD) yielded higher return rates than printed forms (ABILHAND), highlighting the need for user-friendly, digital data collection tools in telehealth studies. These findings suggest that for broader implementation, telehealth programs must pair technology with adequate internet access, training, electronic resources and troubleshooting support.

### Limitations

This study aimed to assess the feasibility of a parent-enacted, remotely delivered intensive pCIMT intervention. It is not possible to draw conclusions about intervention efficacy from this study. In addition, although the interview guide included questions about the remote assessment, parent responses were largely focused on treatment. The assessments chosen appeared appropriate for telehealth data collection but issues with consistency, compliance, and technology impacted the ability to collect pilot data. More research is needed to develop a robust battery of assessments suitable for telehealth. The study also relied on subjective report for compliance with splint wear and asynchronous parent-enacted treatment. Objective tracking of both of these components should be considered in the future. Finally, this study did not measure how well principles of behavioral and motor learning (e.g., operant conditioning, movement repetition, shaping motor behavior, and immediate reinforcement) were present. Subsequent studies may wish to quantify these using measures of intervention fidelity.

### Future Directions

A larger efficacy trial should consider several important design elements: 1) active recruitment of diverse populations to enhance the generalizability of the findings; 2) in-person assessments to accurately measure changes in upper extremity motor function; 3) initial parent training sessions focused on motor and behavioral learning, in addition to within-session instruction; and 4) measurement of intervention fidelity for both synchronous and asynchronous components to ensure consistent and effective delivery. Planning and communication are integral to CHAMP-T's success, and future programs could improve using the parents'suggestions outlined above.

## Supplementary Information

Below is the link to the electronic supplementary material.

Supplementary file1 (DOCX 24 KB)

## Data Availability

No datasets were generated or analysed during the current study.
