# Supplementary material for: Exploring Parent Perspectives of Feasibility After Intensive Telerehabilitation for Children with Unilateral Cerebral Palsy: A Mixed Methods Study
Source: J Dev Phys Disabil. 2025 Sep 1;38(3):543–62. doi: 10.1007/s10882-025-10033-9 (PMC13219209; doi:10.1007/s10882-025-10033-9)
Supplement: Supplementary file 1 — Supplementary file1 (DOCX 24 KB) [file 10882_2025_10033_MOESM1_ESM.docx]

Supplementary File 1: CHAMP-T Parent Interview Questions

1. Was the remote assessment easy to complete? Was it easy to do therapy remotely?

Example probe: what would have made it easier? what made it easy? What challenges did you experience?

2. Did the remote assessment/remote treatment work for you?

Example probe: Was it feasible to complete all the tasks remotely? Was it feasible to gather usable data remotely?

3. Do you think the remote assessment/remote treatment is implementable more broadly?

Example probe: What are barriers to implementing the remote assessment/treatment? What are the strengths of this type of assessment/treatment? What are the benefits of this type of assessment/treatment?

4. Do you think remote assessment/treatment is possible for most therapists/parents?

Example probe: Why? Why not?

5. What are some distinct advantages of remote assessment/remote treatment delivery for families? For therapists and care providers?

Example probe: ask questions about families in particular situations: eg people whose work interferes with their ability to attend therapy in a clinic; people who live in rural settings; people facing bias or discrimination; people who are financially disadvantaged

6. What are some distinct disadvantages of remote assessment/remote delivery for families? For care providers?

Example probe: ask questions about families in particular situations: eg people whose work interferes with their ability to attend therapy in a clinic; people who live in rural settings; people facing bias or discrimination; people who are financially disadvantaged

7. Do you think remote assessment/treatment is an appealing option for families and providers? Can you elaborate?

Example probe: Tell me what makes this appealing. Tell me more about how to make it more appealing.

8. Did you like the remote delivery of the assessment/treatment?

Example probe: What other suggestions do you have for improving the remote delivery?

9. Do you think this type of remote delivery of assessment/treatment would be sought after by families and therapists?

Example probe: Can you expand on why or why not?

10. Do you think it is a good idea to offer remote delivery of assessment and treatment to families as an option for receiving treatment.

Example probe: If not, why? If yes, why?

11. Do you think this type of therapy is a good match for remote delivery?

Example probe: If not, what would need to change? What is the challenge? If so, can you explain what aspects of this type of delivery work well with CIMT.

12. Is IACQUIRE suitable for remote delivery?

Example probe: If not, why? If yes, why?

13. What was the best part of remote assessment/treatment?

Example probe: what worked particularly well?

14. What was the most challenging part of remote assessment/delivery?

Example probe: were there any particular stressors associated with this type of therapy and the way it was delivered? What would you recommend to address these challenges?
